# A political economy analysis of decision-making on natural disaster preparedness in Kenya

**DOI:** 10.4102/jamba.v10i1.497

**Published:** 2018-04-12

**Authors:** Karen C. Rono-Bett

**Affiliations:** 1 Development Initiatives International, Nairobi, Kenya

## Abstract

Most deaths from natural disasters occur in low- or middle-income countries; among them, countries in the Horn of Africa – where Kenya lies. Between September 2015 and September 2016, 23.4 million people in this region faced food insecurity because of the 2015 El Niño, characterised by floods and droughts. The importance of effective government decision-making on preparedness and response are critical to saving lives during such disasters. But this decision-making process occurs in a political context which is marred by uncertainty with other factors at play. Yet, good practice requires making investments on a ‘no-regrets’ basis. This article looks at the factors influencing Kenya’s decision-making process for natural disasters, the preparedness for the 2015 El Niño as a case study. I explored what stakeholders understand by ‘no-regrets investments’ and its application. I assessed financial allocations by government and donors to disaster preparedness. Based on key informant interviews, focus group discussions and financial analyses, this article presents evidence at national and subnational levels. The findings indicate that in making decisions relating to preparedness, the government seeks information primarily from sources it trusts – other government departments, its communities and the media. With no existing legal frameworks guiding Kenya’s disaster preparedness, the coordination of preparedness is not strong. It appears that there is a lack of political will to prioritise these frameworks. The no-regrets approach is applied predominantly by non-state actors. Because there have been ‘non-events’ in the past, government has become overcautious in committing resources on a no-regrets basis. Government allocation to preparedness exceeds donor funding by almost tenfold.

## Introduction

Natural disasters have long-term effects on poor people. According to the 2015 report of the Centre for Research on the Epidemiology of Disasters, ‘The Human Cost of Natural Disasters’ over the last 20 years, over 1.3 million lives have been lost due to natural disasters worldwide. More than half of these deaths were caused by earthquakes, and the remainder were as a result of weather-related hazards such as floods and droughts. The majority of these deaths occurred in low-income (46.6%) and middle-income (31%) countries.

The Horn of Africa and the African Great Lakes region,^1^ where Kenya lies, faces a mix of risks, both natural and man-made, ranging from weather-related natural hazards, conflict and political instabilities and economic shocks (United Nations Office for the Coordination of Humanitarian Affairs [UNOCHA] 2016a).

Between September 2015 and September 2016, the number of people living in food insecurity in the region doubled to 23.4 million.

Despite Kenya’s status as a growing economy and the regional hub for major humanitarian activities, it is still highly vulnerable to the impact of natural disasters. These are mainly drought and flooding resulting in high levels of food insecurity, malnutrition and disease outbreaks. The most affected areas are the arid and semi-arid lands (ASALs) that cover 23 of the 47 counties and comprise about 89% of Kenya’s land mass (UNOCHA 2016a). The most recent flooding was the El Niño event in 2015. By the end of that year, floods had affected an estimated 35 565 households, with 12 398 households forced into displacement. The floods also resulted in a loss of animals and agricultural crops (Kenya Red Cross 2015).

With this level of vulnerability, and yet a growing economy, I question what factors contribute to the country remaining vulnerable to disasters. I posit that this is likely to lie with the decision-making process, which in turn is affected by the political economy. Past literature indicates that disasters occur in a political space (Cohen 2008). By political economy, I mean the availability and use of information; existing institutions and their relationships; legal and governance frameworks in place; incentives and disincentives for decision-making; as well as the financial resources that are available and allocated. Good practice guides that decisions and investments on preparedness should be made on a no-regrets basis – that is, investments should be made whether the disaster occurs or not as the net benefits outweigh the costs. This will provide resilience and therefore reduce the vulnerabilities of populations exposed to such disasters.

With this political economy approach, and focusing on the 2015 El Niño event as a case in point, I seek to understand the following:

- What are the factors that influence decision-making regarding disaster preparedness in Kenya?
- What is understood by no-regrets investments and how is this applied in Kenya?
- How much is allocated to Kenya’s disaster preparedness at the national and subnational level?

**FIGURE 1:**
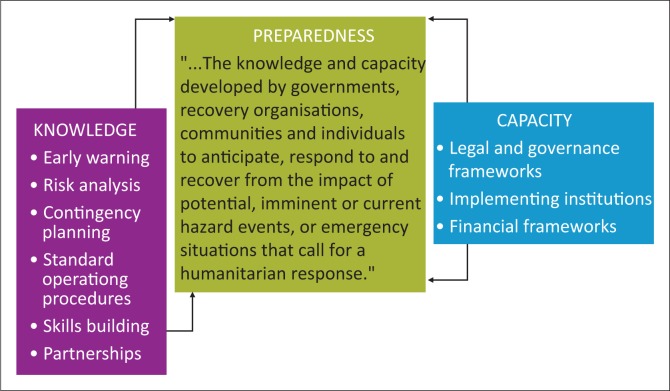
The construct of disaster preparedness.

This study uses the definition of disaster preparedness proposed by the United Nations International Strategy for Disaster Reduction (UNISDR) and UNOCHA and proposes a construct for disaster preparedness decision-making (Figure 1). Preparedness, in the context of disaster risk management, focuses on building capacity to ensure the efficient management of emergencies and building sustainable systems for resilience and recovery.

### Methodology^2^

The researcher carried out key informant interviews at the national and subnational level (Mandera and Migori counties). These two counties were among those affected by flooding and disease outbreaks during the 2015 El Niño.^3^ Focus group discussions with communities affected by disasters were used to understand the barriers and enablers to disaster preparedness at a subnational level. Last, the study analysed resources allocated to disaster preparedness through aid and domestic budgets. These approaches were applied to triangulate findings and generate both quantitative and qualitative information for analysis. Key informants’ quotes are shown in quotes; and I only attribute the type of institutions they represent, for example, government, legislature or an international non-governmental organisation (NGO), unless in situations where I sought permissions.

In the next sections, I present a review of literature and then the results of the study. The article wraps up with some concluding remarks.

## Literature review: Decision-making in a disaster context and the concept of no-regrets investments

### The challenge of decision-making in a disaster context

Decisions on preparedness actions and the subsequent allocation of resources are often driven by the political economy – the institutions and individuals where power lies, the incentives for different types of behaviour and the drivers that govern or influence decision-making. Preparedness should be guided by information on risk analysis and early warning. Contingency plans and standard operating procedures need to be in place to guide decision-making and resource allocation. Institutions with the proper legal and financial frameworks should back preparedness (UNISDR 2007).

The level, or lack thereof, of government preparedness and response, and the speed of its decision-making, will influence the extent to which populations are affected by disasters – this means that disasters always happen ‘in a political space’ (Cohen 2008).

The context of a natural disaster is, however, inimical to speed in decision-making. Humanitarian actors – often with limited information and time – need to make decisions that accommodate different perspectives and organisations (ALNAP 2016). Nonetheless, decisions are expected to be, in as much as it is possible, cost-effective, efficient and made with ‘no-regrets’ (Oxfam and Save the Children 2012).

### No-regrets approach in the disaster context

No-regrets commitments are ‘actions by households, communities and local/national/international institutions that can be justified from economic, and social, and environmental perspectives whether hazards take place or not’ (Siegel 2011:2). Such commitments refer to measures that are enacted without certainty about the probability of the occurrence of a natural disaster (or indeed its magnitude); the commitments therefore provide resilience to the natural disaster and reduce the vulnerabilities of populations exposed to such events.

No-regrets investment is based on the theory that investing based on risks and a certain level of uncertainty will result in net positive effects in the long term, even if the anticipated risk does not materialise and costs are incurred in the short term. For this reason, it is regarded as anticipatory and cost-effective.

Consequently, the importance of disaster preparedness and early action is referenced in policy documents and commitments. For example, the UK Government’s 2011 Humanitarian Emergency Response Review identified disaster resilience and placed it at the centre of its approach to addressing disasters (Venton 2012).

More recently, the UN Secretary General’s report for the 2016 World Humanitarian Summit identified the importance of investing early and sustainably, even where donors are not rewarded with domestic and international visibility. It states that ‘resources should be disbursed on a “no-regrets” basis and support provided to interventions that deliver benefit whether or not the anticipated risk materialises, such as stockpiling relief supplies’ (United Nations 2016:40). It also states that financial incentives to reward risk-informed local and national early action should be developed.

According to Venton (2012), disaster preparedness would save between $107 million and $167m for a population of 367 000 in Kenya, and between $662m and $1.3 billion for a population of 2.8 million in Ethiopia in a single event alone. The wider benefits of building resilience can significantly outweigh the costs involved – for example, the process of drought recovery takes longer when a community is not resilient.

Investments made on a no-regrets basis – even if the disasters do not transpire – will result in a net positive effect in the long term, either because the investment costs are lower or because resilience will be strengthened (International Federation of Red Cross and Red Crescent Societies 2014). Examples of no-regrets efforts include the pre-positioning of stocks; market assessments; early engagement with the private sector to develop standing agreements and with donors to develop response plans; and establishing human resource systems (Oxfam and Save the Children 2012).

Despite the long-term net benefits, there will be aspects of early action that have no benefit in the short term. For example, incurring storage costs when the increased demand does not materialise. The no-regrets approach is based on the theory that incurring such costs is acceptable, given that the value of the preparedness achieved makes the cost of an occasional non-event acceptable (World Vision 2014). In summary, the concept of no-regrets investments is an overall principle for decision-making on early action rather than a standalone funding tool (Siegel 2011). The no-regrets approach relates specifically to mobilising flexible funding mechanisms (Food Security and Nutrition Working Group 2013) and goes beyond establishing systems for early warning – it informs the decision to act. This requires identifying and agreeing on the triggers for no-regrets investments (Oxfam and Save the Children 2012).

While no-regrets investments are seen as good decisions, not much is understood of the concept and its application in a context like Kenya.

## Results

### Factors that influence decision-making on disaster preparedness in Kenya

#### The availability and use of information and evidence

Almost no respondents linked the lack of capacity to prepare for disaster to a paucity of evidence or information. Several sources of data on disasters, risks and weather information exist; in fact, the country reports the highest number of data sets (355) on the Humanitarian Data Exchange (2016). Various systems such as the Famine and Early Warning Systems Network (FEWSNET), the INFORM Index for Risk Management and weather predictions from the Department of Meteorological Services help to close the data gaps.

Previous studies suggest that humanitarian actors working under time and information constraints often rely on information from sources they trust to make decisions (Development Initiatives 2016; ODI 2009). A study in Ethiopia found that effective action depended on the quality of relationships with government decision makers, the use of informal networks, a good understanding of the government system and trust (Darcy et al. 2013). This shows that the availability of accurate early warning data is not the only determinant in government decision-making processes – and I found the same in Kenya. Development Initiatives’ 2016 study on humanitarian-systems mapping in East Africa, which highlighted the use of evidence in the humanitarian sector, found that trust plays a role on the uptake of evidence on two levels. First, it influences the ability of decision makers to value and understand information; and second, the limited engagement between humanitarians and government policymakers has limited the level of trust on information produced (Development Initiatives 2016).

This study found that government decision makers and members of the community use trust and a variety of media to access information. In particular, government ministries, community meetings, telephone conversations, local radio stations, word of mouth and the police were cited as the most useful sources of information:

‘I rely on the information from my constituents, when there is something happening on the ground, they call me immediately and I am able to travel there to help in resolving the matter.’ (Member of Parliament, female, former humanitarian expert, pers. comm., 12 August 2016)

Information from community members, while not always formal, indicates the importance of trust for promoting the uptake of evidence. This finding matches findings in Ethiopia where relationship and trust building with government decision makers is equally important to evidence in decision-making (Darcy et al. 2013).

When asked where they go for documented evidence, respondents from government agencies indicated they rely on information from their own respective ministries. For flood or weather information, for example, they go to the Ministry of Water and Irrigation, the Water Resources Management Authority or the Department of Meteorological Services. Respondents from international NGOs tend to rely on evidence from other humanitarian actors such as FEWSNET. These responses match the findings of the humanitarian evidence systems mapping in East Africa (Development Initiatives 2016) – that people trust information from their own networks:

‘The Kenya Meteorological Department is our main source of weather information.’ (Government Agency, male, Deputy-Director, pers. comm., 25 July 2016)

In 2015, it was only after the Department of Meteorological Services announced the onset of El Niño that the government took action and mobilised a response – despite other organisations having shared information about the coming floods.

#### A lack of political will and a culture of preparedness – with the legal and governance frameworks not fully operational

Because Kenya has sufficient data that could inform government decision-making, the question arises as to whether it lacks the political will or a culture of preparedness. There are some paradoxes.

First, while Kenya has committed to international disaster preparedness frameworks, such as the Sendai Framework for Disaster Risk Reduction and Hyogo Framework for Action, a lack of political will among government decision makers hinders the consideration of disaster preparedness as a national priority (Government of Kenya 2016). This is evidenced in the slow progress in putting in place legislation on disaster preparedness.

Second, Kenya was the first country in Africa to join the Africa Risk Capacity (Africa’s first sovereign catastrophe insurance pool) in 2012. Since 2014, it has paid an annual premium of approximately $9m (around KES 900 million). Despite this investment, an awareness of the Africa Risk Capacity was very limited, with fewer than half of the respondents aware of Kenya’s decision to participate.

As shown in Figure 1, preparedness requires legal and governance frameworks to guide decision-making. Kenya does not have a law that guides its disaster preparedness operations:

‘Without a law, accountability for disaster response goes down.’ (Government Agency, male, head of agency, pers. comm., 12 July 2016)

Respondents reported that there is just not enough pressure or incentives on government to pass the law:

‘There is not enough loud voice on it. To pass the law does not require normal workshops it needs more lobbying. Media engagement is also needed.’ (Member of Parliament, female, former humanitarian expert, pers. comm., 12 August 2016)

**Institutions responsible for disaster preparedness in Kenya:** A process of putting a law in place started in 1999 and has not been completed to date. This has led to disaster preparedness being fragmented and duplicative. Three government institutions lead the country’s disaster preparedness. The National Disaster Operations Centre was established in 1997 and is responsible for coordinating all disaster response operations in the country. It led the country’s El Niño flood response in 2015. The National Disaster Management Unit is responsible for disaster risk management. Led by the National Police Service, the Unit also carries out response activities (National Disaster Management Unit n.d.). It has established the country’s emergency response plan and standard operating procedures though study respondents, Non-state actors particularly, have had little interaction with the Unit and many were not aware of the plans and standard operating procedures.

The third institution is the National Drought Management Authority. This was established in 2011 and plays a leading role in drought preparedness and response in the ASALs (National Drought Management Authority n.d.). It has two coordination bodies at the national level, bringing together various stakeholders in drought preparedness. These are the Kenya Food Security Meeting and the Kenya Food Security Steering Group. The National Drought Management Authority is highly regarded by respondents because of its successes in reducing deaths from droughts in the country, and because of its presence in the ASAL counties, supporting drought preparedness.

Respondents in this study did not see the value of having three institutions carrying out similar activities on disaster preparedness, stating that this leads to a duplication of efforts and internal competition between these institutions.

**National and subnational relations towards disaster preparedness:** The Kenyan Constitution, which introduced the devolved form of governance, assigns the responsibility of disaster preparedness and management to both the national government and the county governments. However, currently there seems to be no standard and no clearly defined disaster preparedness goals that all counties can commit to. This is in part because of the lack of a disaster preparedness law at a national level and in part because of the shared function outlined in the constitution. Counties have taken the initiative to self-organise, at times with a limited knowledge of disaster preparedness or the ability to align their disaster preparedness plans to national or global processes. However, ASAL counties like Mandera have benefitted from the National Drought Management Authority’s engagement and developed more systematic approaches to disaster preparedness, unlike non-ASAL counties such as Migori, which do not have this guidance from an institution at the national level.

Across the counties, where disaster preparedness is positioned and how it is conceptualised varies. In Mandera, it is positioned under the Ministry of Public Service, while in Migori it is under the Ministry of Environment, Natural Resources and Disaster Management. This lack of harmonisation plays a role in affecting response and coordination across the country and counties. Interviews with respondents showed that out of the 47 counties, only 4 (Baringo, Kisii, Tana River and Nairobi) have disaster preparedness laws already passed by their respective county assemblies.^4^

#### Effect of disaster non-events and the importance of cultural factors

Kenya has experienced two El Niño ‘non-events’ in the recent past. Non-events mean disasters occurr but to a lesser magnitude than was predicted. Disaster non-events have affected government decision makers and limits no-regrets investment decisions.^5^ The 2015 El Niño was declared a national emergency only a month before the onset of the rains. The decision was made only after the Department of Meteorological Services announced the onset of El Niño, despite earlier declarations from other early warning systems (UNOCHA 2015). The decision to delay the announcement was because of previous non-events; these guided the government not to declare a disaster until it was sure. Nevertheless, this late announcement limited the amount of time that various humanitarian actors had in which to make decisions, prepare and respond. According to UNOCHA, had government made the announcement much earlier, the country would have averted the loss of property and lives (UNOCHA 2016b).

The non-events have affected communities differently. In Migori County, which is less prone to disasters, the non-events have negatively affected local communities’ willingness to prepare for disasters. For example, in the lowlands of Migori County, people are well aware of the effects of floods, but often are not quick enough to prepare or to respond to information. This is linked to previous disaster predictions not materialising, and cultural and economic factors, notably the inability to recover fully from the effects of disasters (Migori County, male, community worker, pers. comm., 28 July 2016). In Mandera County, however, which is more prone to disasters, the need to prepare for future disasters has not changed among the county stakeholders following the non-events. In fact, communities have established contingency plans.

For Mandera County, religion and traditional practices play a part in the communities’ perceptions of non-events. On the positive side, there is the general belief that everything happens only with God’s permission. As such, there is better preparedness with an incorporation of local knowledge and practices. On the negative side, religion can play a role in ‘non-preparedness’. Because people believe their fate is in the hands of a higher power, they believe that whatever they do might not have an effect on the outcome of God’s will:

‘Among us Muslims, non-events are not a surprise. We believe that while man uses science to predict, the Will of God plays a bigger role. We believe God has given man the knowledge to plan and to execute plans but ultimately, God is the final decider.’ (Mandera County, male, community member, pers. comm., 09 August 2016)

### Applying the no-regrets principle in-country

The principle of decision-making on a no-regrets basis is viewed as good practice in literature (United Nations 2016). The study found more application of the no-regrets approach among non-state actors than with government decision makers. For example, non-state actors in the donor community used the approach to plan and respond to the 2015 El Niño. The investments included pre-positioning food rations for refugee camps, supporting the purchase of livestock vaccines and increasing cash transfer allocations to reach additional households that were likely to be affected by the disaster.

At the subnational level, most development organisations operating in Mandera County allocate resources on a no-regrets basis.^6^ This is in the form of pre-positioning, market assessments, developing early warning systems and building the resilience of their beneficiaries. For these organisations, decision-making regarding resource mobilisation and investments is influenced by both community priorities and donors’ thematic interests.

There are variations in terminology on (what is termed in this article) no-regrets investments. The academia in Kenya, for example, refers to ‘starter activities’. World Vision applies the principle through contingency funding known as ‘national disaster preparedness funds’ that have an agreed proportion of development funds allocated for emergencies and are activated within 24 hours of a disaster occurring (WorldVision, female, manager, pers. comm., 08 August 2016).

Despite signs of the application of the principle among non-state actors, there is no consensus around the activities that could be regarded as no-regrets. In 2014, a consultation process among international humanitarian NGOs, led by the International Federation of Red Cross and Red Crescent Societies to seek consensus around key activities that could be seen as no-regrets, did not reach full consensus.

Participants in the 2014 consultation process agreed on activities that were no-regrets investments and those where there was no consensus, respectively (Tables 1 and 2).

**TABLE 1:** Examples of activities agreed to be no-regrets investments.

Number	Examples
1	Training for resources management committees
2	Hygiene awareness and pre-positioning of hygiene kits
3	Support to community contingency funds
4	Pre-identification of possible sources of funds for early action
5	Mapping and contractual agreement preparation with financial institutions
6	Revision of contingency plans
7	Identification of potential zones at risk
8	Pre-crisis markets mapping and analysis
9	Mapping of government and partners plans in relation to a threat or warning
10	Production and dissemination of guidance to partners

**TABLE 2:** Examples of activities not agreed to be no-regrets investments.

Number	Examples
1	Livestock mass vaccination
2	Continued commercial destocking
3	Food vouchers
4	Non-food items distribution
5	Unconditional cash transfers
6	Scale-up level of unconditional cash transfers
7	Scale-out coverage of unconditional cash transfers
8	Preparedness for a cash response through printing of vouchers
9	Humanitarian staff recruitment

The principle of decision-making on a no-regrets basis is viewed as good practice in literature (United Nations 2016). This study found that the application of a no-regrets approach was more common among non-state actors than with government decision makers:^7^

‘Emergency items are often consumables, there is less accountability and hence this is often preferred.’ (Government Respondent 2016)

### Resource allocation to disaster preparedness

Disaster preparedness is projected to save up to $7.00 for every dollar that is invested (UNDP 2012). At present, investments in preparedness are modest compared with allocations to emergency response; however, this could be changing.

#### Donor funding to disaster preparedness

**FIGURE 2:**
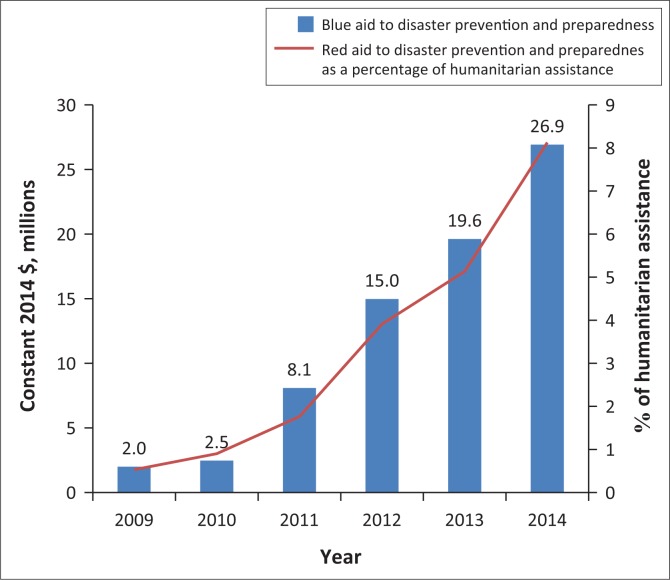
Donor funding to disaster prevention and preparedness between 2009 and 2014.

Donor funding to Kenya for disaster prevention and preparedness, as reported by the Organisation for Economic Co-operation and Development, has increased more than tenfold between 2009 ($2m) and 2014 ($26.9m). However, this allocation remains small compared with total humanitarian assistance to the country, making up less than 10% of the total (Figure 2).

### Domestic budget allocation to disaster preparedness

**FIGURE 3:**
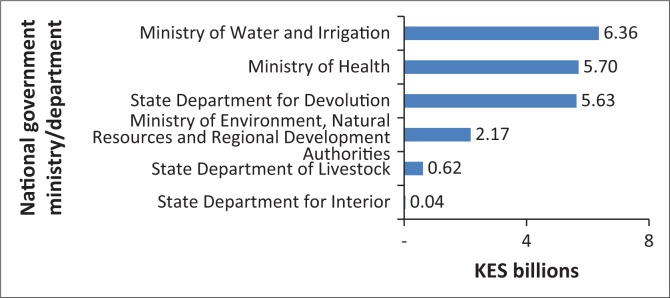
Domestic funding to disaster preparedness financial year 2016–2017.

In the 2016–2017 budget, allocations for disaster preparedness projects formed an estimated 2.5% (KES 20.5 billion – $205m) of Kenya’s total budget (Figure 3). This is about 10 times the amount of funding the country received from donors for disaster preparedness in 2014.

While resources are allocated to disaster preparedness, it is not known if these are sufficient to meet the country’s preparedness needs, if these resources are allocated to the highest priority sectors and whether the government could indeed be making no-regrets investments already.

## Conclusion

This study of the political economy of decision-making for disaster preparedness in Kenya presents findings that can inform change and promote the political will towards better decisions on preparedness actions and the allocation of resources to them.

Trust (and relationships) plays an important role in decisions and action. Given the limited time that decision makers have, they rely on information from specific sources that they trust – mostly their own – and use this to guide their decision-making (Development Initiatives 2016; Oliver 2014). In Kenya, government decision makers trust information not only from government but also from their community members and the local media.

Humanitarian actors should put efforts towards ensuring that accurate and timely information is available through these mediums in order for change to happen. If passed on at the right time, early warning information using channels that reach the communities has a high likelihood of informing government decision makers in order to translate to early action and better disaster preparedness.

Kenya has sufficient data that could be harnessed to inform decision-making. I find similar patterns in Ethiopia (Darcy et al. 2013). The study concludes that it is the lack of political will that leads to the insufficient use of data in decision-making.

Humanitarian actors in Kenya need to invest in relationship building with government decision makers. This will result in better decision-making. Ultimately they need to promote the further use of data to inform decision-making.

Kenya does not have well-established legal and governance systems that guide decision-making on preparedness actions. There is no disaster risk management law. The lack of such legal systems affects accountability. At present, the governance of disaster preparedness and response sits in three government institutions, which, if not clearly defined, could pose a problem. The effectiveness of the country’s disaster preparedness structures – governance, financial and policy – are challenged even more with the context of devolution, given that disaster preparedness is a shared function between national and county government. A useful recommendation is for the Government of Kenya to fast-track the passing of the disaster risk management law to bring more clarity on roles and responsibilities around disaster preparedness and how the various institutions should interact and function at the national and county level.

No-regrets investment is an important approach to disaster preparedness. However, decisions to make no-regrets investments are largely influenced by differences in people’s awareness of it and the context in which it is applied. Humanitarian actors understand this terminology differently. Donors and international organisations use the term and appear to practice it much more than local actors. Donors and international actors, therefore, need to build a consensus on no-regrets investments and cascade this approach to local and government actors at the country and county level. This is important in building the resilience of communities and preventing disasters from escalating. In addition, a no-regrets approach is also useful for promoting development and poverty reduction.

Allocations to disaster preparedness are forecast to save up to $7.00 for every $1.00 spent. In Kenya, the domestic resource allocation to preparedness exceeds donor funding to preparedness almost tenfold. However, not much is known about whether the resources are allocated efficiently and on a no-regrets basis.

As an area for further research, more analysis is needed to understand government decisions on resource allocation, if these are allocated to sectors with the most need and if the spending already meets the no-regrets criteria. More analysis is also needed to understand the current funding gap and the amount of funding that is required for preventing disasters.

Lastly, non-events have negatively affected the perception of the need and urgency for preparedness, particularly in locations that are less prone to disasters. This has likely affected government decision-making regarding disaster preparedness. This limits making investments on a no-regrets basis. In addition, communities in these locations, which are less prone to disasters, are less likely to prepare for disasters because of previous non-events. This study finds that the perception of the need to prepare has not been affected by the non-events in areas that are more prone to disasters, such as Mandera County.

A useful recommendation is the active promotion of disaster preparedness to government decision makers and communities, as a more cost-effective, long-term solution to averting disaster compared with response. It is only until this is appreciated that non-events will not affect perceptions of the need to prepare for disasters.

## Footnotes

1 The countries are Ethiopia, South Sudan, Sudan, Kenya, Burundi, Rwanda, Uganda, Eritrea, Djibouti and Somalia.

2 Some of the limitations for this study are the following: the concept and measure of no-regrets investments could be improved in future studies, to better assess the level of preparedness through financial commitments in a state of uncertainty. More interestingly, the power interplay between actors involved and their use of information or data in decision-making would benefit from replications of this type of study.

3 Migori County lies in the southwestern part of Kenya, bordering Tanzania and Lake Victoria. With a population of 0.9 million, almost half of the people (48.4%) live below the national poverty line. The county ranks fourth on exposure to floods. Mandera County is in the northeastern part of Kenya that borders Ethiopia and Somalia. With a population of 1.02 million, 85.8% of the people live below the national poverty line. The county is classified as an ASAL, with scanty and unpredictable rainfall patterns. Nomadic pastoralism is the main economic activity, with the residents rearing camels, goats, sheep and cattle. Mandera County is a marginalised community, physically exposed to drought.

4 Members of the County Assembly are the policymakers at the county level.

5 I take this as anecdotal evidence because I do not analyse government spending on previous non-events to conclude that they do not make investments on a no-regrets basis.

6 Such organisations include the Agency for Technical Cooperation and Development, the Norwegian Refugee Council, Islamic Relief, and local organisations such as the Rural Agency for Community Development and Assistance.

7 Some of the questions in the key informant interview included the following: ‘Does your organisation make no-regrets investments in preparedness activities? What influences your decisions to make these investments?’ International NGOs interviewed were able to respond in the affirmative and provide examples of how they make no-regrets investments. Government respondents were aware of the concept but admitted that it was always easier and faster to make response decisions rather than preparedness decisions. A further analysis of the budget allocations can help to prove if this is true. There could, however, be some no-regrets investments within the government.
