# Erratum: A political economy analysis of decision-making on natural disaster preparedness in Kenya

**DOI:** 10.4102/jamba.v10i1.722

**Published:** 2018-08-15

**Authors:** Karen C. Rono-Bett

**Affiliations:** 1 Development Initiatives International, Nairobi, Kenya

In the version of this article published earlier, the ‘Acknowledgement’ section contained the incorrect display of the funder’s full name as ‘Department for Internal Development (DfID)’, which has been corrected to ‘Department for International Development (DfID)’. The full ‘Acknowledgement’ statement is hereby updated and replaced with:

